# Comorbidity risk and distribution characteristics of chronic diseases in the elderly population in China

**DOI:** 10.1186/s12889-024-17855-w

**Published:** 2024-02-03

**Authors:** Zihang Xiang, Hao Wang, Handong Li

**Affiliations:** 1 School of Systems Science, Beijing Normal University, Beijing, 100875 China; 2 China National Health Development Research Center, Beijing, 100044 China

**Keywords:** Elderly, Chronic diseases, Comorbidity risk, Complex network, Multistate transition model

## Abstract

**Background:**

The risk of comorbid chronic diseases in elderly people is an important problem affecting their health and quality of life. We analyzed the incidence of chronic diseases for combinations of chronic diseases analyzed.

**Methods:**

We used the original data to construct hypothetical cohorts of elderly individuals that evolved with age. The complex network was used to reduce the dimensionality of disease. The multistate transition model is used to calculate the incidence of each chronic disease, exploring comorbidity characteristics and rules.

**Results:**

(1) By using complex network, seven chronic diseases were screened out in men, including hypertension, diabetes, heart disease, stroke, chronic lung disease, arthritis and dyslipidemia; six chronic diseases in women showed significant comorbidity except chronic lung disease. (2) Incidence show differences in age and sex; incidence of chronic diseases generally increased with age. (3) The marginal risk increases with the number of basic chronic diseases associated with comorbidities. (4) When hypertension is present as a basic disease, its impact on the risk of other chronic diseases is much less than that of other chronic diseases. (5) When diseases occur as basic chronic diseases, hypertension–heart disease and diabetes–dyslipidemia are combinations that have the greatest impact on each other in men; hypertension–heart disease in women.

**Conclusions:**

The incidence of chronic diseases in patients who have chronic diseases and will form comorbidities differs from that in healthy states, and the related effects of different chronic diseases also differ. Among these conditions, hypertension is caused by a special mechanism.

## Background

### Introduction

According to China's seventh population census, the number of elderly people aged > 60 years in China reached 264 million in 2020, accounting for 18.7% of the total population, and the proportion of elderly people suffering from various types of chronic diseases reached 20% [1]. As the Chinese population ages, the proportion of the elderly population suffering from various chronic diseases also gradually increases. Chronic diseases, which have a long duration and, theoretically, cannot be cured and solved, constitute a large part of the medical problems regarding the elderly population. The disease burden of chronic diseases also reached more than 70% of all diseases by 2019 [2]. The Global Health Statistics Report 2017 published by the World Health Organization (WHO) emphasized that chronic diseases cause more than 41 million deaths worldwide, accounting for 71.3% of the total number of deaths [3].

The World Health Organization defines the phenomenon in which individuals suffer from two or more chronic diseases as comorbidities [4]. Comorbidity is a state in which multiple diseases coexist. When an individual already has a chronic disease, the relapse of any other, unaffected chronic disease will lead to the emergence of new comorbidities. Existing studies are mostly based on the risk of a single chronic disease, while the study of comorbidities or chronic disease risk associated with comorbidities has more general significance.

### Literature review

As the aging of China's population accelerates and the resulting health problems become increasingly prominent, studies have gradually paid increasing attention to the incidence of chronic diseases. Research on the incidence of chronic diseases and comorbidities has been divided into two main steps: First, research has mainly focused on the incidence of chronic diseases based on the dynamic data of each hospital and has conducted certain statistical analyses and analyses of the main factors affecting the incidence; second, research has focused on the comorbidity of chronic diseases based on demographic data, analyzed the comorbidity patterns of major chronic diseases, and used methods such as correlation and clustering to identify diseases with similar comorbidity patterns.

At present, most of the research elaborate on the prevalence through statistical analysis and summarize the prevalence characteristics of a certain area for single or multiple chronic diseases. Siyu Li et al. collected monitoring data on four types of chronic diseases, namely, diabetes, stroke, acute coronary heart disease events and malignant tumors, in Taizhou city, Zhejiang Province and obtained the incidence from medical dynamic data [5]. Jiehong Jin et al. analyzed the incidence of chronic diseases such as malignant tumors, stroke, diabetes and coronary heart disease in Dongyang city from 2009 to 2015 using descriptive epidemiological methods [6]. Nan Chen et al. conducted a summary analysis of eight chronic diseases, such as tumors and coronary heart disease, based on annual statistics of noncommunicable diseases in Hedong District, Tianjin, from 2011 to 2015. The authors found that the incidence of these eight chronic diseases increased annually, and the incidence was greater in men than in women [7]. Yan Wang et al. used case reports of five chronic diseases, such as essential hypertension and stroke, in Daqing city from 2004 to 2013 to describe the incidence trends of chronic diseases and calculated the sex- and age-specific incidence of each disease [8]. Huimin Jin et al. selected various physical indicators obtained from the physical examination of a certain school staff member as influencing factors and observed their correlation with chronic diseases such as hypertension and hyperlipidemia. They found that there were significant differences in sex in patients with diseases such as hypertension and hyperlipidemia. Specifically, the correlation is stronger in men that women [9]. Van Oostrom SH et al. analyzed the incidence of chronic diseases in the Netherlands overtime and conducted factor regression analysis [10].

With the accumulation of chronic diseases, the number of elderly patients with comorbid chronic diseases in China is continuing to increase. In recent years, correlation research on comorbidities in the elderly has become a hot topic. Using the CHARLS dataset, Jinjia Lai et al. explored the associations between health-related behaviors and chronic disease comorbidities in middle-aged and elderly people through an association analysis algorithm [11]. Hao Xu et al. used Nanjing's chronic disease surveillance data from 2017 to 2018, adopted a multistage stratified random sampling method to obtain samples, and used a generalized linear mixed model to fit multilevel logistic regression analysis to explore the comorbidity status of hypertension, diabetes and dyslipidemia [12].

An important aspect of comorbidity research is to focus on one or more chronic diseases and explore the comorbidity status and comorbidity patterns of these chronic diseases and other diseases. Based on data from tertiary hospitals in Henan Province, Qianqian Hu et al. explored the correlation between comorbidities and ischemic stroke incidence and used a cluster analysis method to explore their comorbidity patterns [13]. Derong Peng et al. used association rule analysis to analyze the associations between comorbidities and various diseases in hypertension patients in Shanghai communities, and they found that comorbidities were strongly associated with different numbers of comorbidities [14]. Ye Pan et al. used four methods, namely, association rule analysis, cluster analysis, principal component analysis and potential category analysis, to explore the comorbidity patterns of elderly people in China and revealed a consistent comorbidity pattern through comparative analysis [15]. Chunzi Cui et al. used systematic cluster analysis and the Apriori algorithm to determine the mode of chronic disease comorbidity [16]. Yanna Li et al. explored the trend of comorbidities by the chi-square test, and the significant associations between chronic diseases were analyzed by network analysis [17].

In foreign studies, Oflaz Zarina et al. combined hidden Markov theory with copula functions and constructed a model to describe the interaction process of two or more comorbidities; this model was verified to be effective on the basis of no clinical medical data [18]. Caldeira, T.C.M. et al. studied the comorbidities of obesity and hypertension or diabetes during the 16-year period from 2006 to 2021 and reported that the incidence of these conditions increased significantly in male and elderly individuals over time [19]. Sun MX et al. studied the prevalence of multiple diseases in the Chinese population, analyzed the effects of different comorbidity modes and their corresponding all-cause mortality risks, and found that the comorbidity combinations associated with the highest incidence were hypertension–chronic kidney disease and hypertension–diabetes–chronic kidney disease, among which the comorbidity combination with diabetes–chronic kidney disease would was associated with the highest mortality risk [20]. Sun, P. et al. established a simulation model of the whole life cycle to characterize disease metastasis within the cycle, built a simulation framework of the whole life cohort on the basis of the probability model of chronic disease transition, and verified the effectiveness of the framework using the original short- and medium-term data [21]. Uddin S et al. used a total of 24 years of administrative medical data from 1995 to 2018 to explore common chronic diseases and their major comorbidities and used network analysis methods to rank comorbidity conditions leading to the onset of different diseases. They found that patients with cardiovascular disease and diabetes had a greater probability of developing the remaining comorbidities that also increased significantly with time and age [22].

The existing studies have the following limitations. First, studies on the incidence of chronic diseases in elderly individuals have been based mainly on pathological data. The existing studies in which the incidence of diseases was obtained through dynamic medical data have few data sources, high access thresholds, small sample sizes and poor universality; moreover, traditional statistical methods are mainly used in these kinds of studies. Second, studies on the calculation and analysis of disease incidence on the basis of disease comorbidities are rare. Most of these studies separate the modes of disease comorbidities through association and clustering but lack calculations of incidence on the basis of disease comorbidities. In some studies, the concepts of disease incidence and incidence are vague, and most studies have focused more on the association between disease prevalence and incidence. Finally, even fewer studies have focused on most chronic diseases at the same time; most studies are based on two or three specific diseases and their comorbidities. The main contributions of this article include the following aspects:

We constructed a chronic disease incidence calculation model based on single cross-sectional data using the hypothetical cohort, complex network was constructed to identify the diseases with the highest frequency among all comorbid conditions and reduce the computational dimension. Then, we used a multistate transition model to calculate the incidence of each chronic disease in the process of identifying a new comorbidity if it became debilitating. We applied the above model on the basis of Chinese Longitudinal Healthy Longevity Survey (CHLHS) data and obtained information on the mutual promotion of major chronic diseases under comorbid conditions. We mainly analyzed the incidence of chronic diseases from two aspects. For each specific target chronic disease, from one perspective, we can see which diseases most significantly affect it. We rank the risk of each chronic disease under other different combinations of chronic diseases; from another perspective, we can analyze how much impact a chronic disease will have on other chronic diseases during the formation of comorbidities. In this perspective, the target disease can be seen as the basic disease in comorbidity. We give the marginal impact and ranking of each chronic disease on other chronic diseases.

The remainder of this article is organized as follows: the next section provides a literature review, and the second section provides an explanation of the model and specific methods used in the article. The third section provides an analysis of the empirical results, the fourth section provides a discussion of related issues, and the conclusion concludes the article.

## Methods

### Overview of methods

**Fig. 1:**
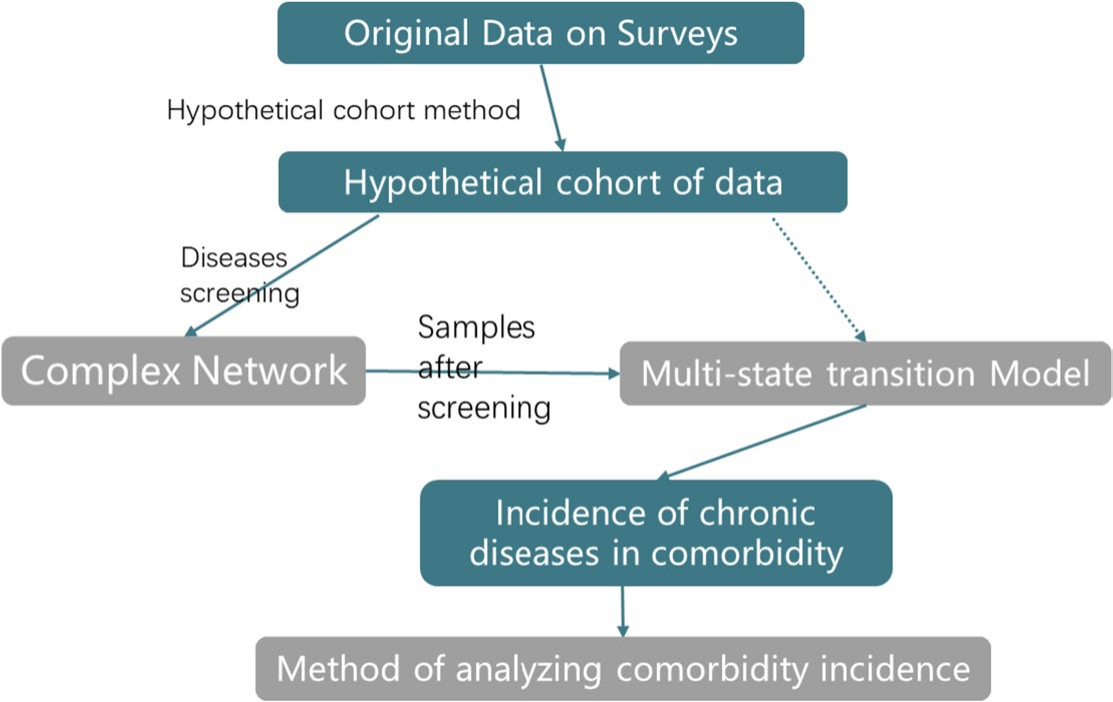
Abridged general view of analyze method

We constructed a set of chronic disease comorbidity risk assessment models based on cross-sectional data. This model first performs data scaling on the original survey sample to obtain a hypothetical cohort in which the incidence of chronic diseases changes with age; then, the complex network method is used to screen the main chronic diseases based on the above normalized data, that is, to reduce the dimensionality of the number of chronic diseases; and finally, the conditional incidence of each chronic disease in the development of various comorbidities is calculated. The model framework is shown in Fig. 1.

### Data reduction

The dataset includes as many older individuals as possible and includes each individual's current chronic disease situation. The CLHLS dataset is a tracking dataset that includes information from elderly people. In addition to death samples, this dataset conducts surveys on the same samples in each survey round, while some new samples are added. The survey covered 23 provinces across the country; 65 and above respondents were included, and abundant samples were collected for people 80 years or older. We obtained complete response data for 16 major chronic diseases from the CLHLS dataset from 2017–2018; a total of 12,139 complete samples were obtained, including 5412 elderly men and 6727 elderly women. A total of 41 age groups ranging from 65 to 105 + years were considered; patients that were 65 ~ 104 years old were divided into groups by year, and people older than 105 years were included in the 105 years old group. The chronic diseases included hypertension, diabetes, heart disease, stroke, chronic lung disease (including bronchitis, emphysema, pneumonia, and asthma), cancer, chronic stomach disease, Parkinson's disease, arthritis, dementia, epilepsy, cholelith disease, dyslipidemia, rheumatism, chronic nephritis, and chronic hepatitis. Table 1 shows the baseline characteristics of the data we used.

**Table 1:** Baseline characteristic in 2017–2018 survey round of CLHLS

Characteristic		number	Proportion (%)
**Sex**	male	5412	44.58%
	female	6727	55.42%
**Age (years old)**	65–69	1530	12.60%
	70–74	1751	14.42%
	75–79	2071	17.06%
	80–84	2203	18.15%
	85–89	1801	14.84%
	90–94	2161	17.80%
	95–99	1377	11.34%
	100–104	2472	20.36%
	105 +	405	3.34%
**Chronic Disease**	hypertension	4805	39.58%
	diabetes	1078	8.88%
	heart disease	1971	16.24%
	stroke	1300	10.71%
	chronic lung disease	1227	10.11%
	cancer	170	1.40%
	chronic stomach disease	539	4.44%
	Parkinson's disease	88	0.72%
	arthritis	1228	10.12%
	dementia	262	2.16%
	epilepsy	29	0.24%
	cholelith disease	463	3.81%
	dyslipidemia	587	4.84%
	rheumatism	591	4.92%
	chronic nephritis	130	1.07%
	chronic hepatitis	51	0.42%

### Construction of the hypothetical cohort

To calculate the probability of chronic disease transition from cross-sectional data, this article used the method of a hypothetical cohort, in order to convert samples of different cohorts into samples of the same cohort, so as to calculate the state transition probability with age, and smooth the data.

First, the age-specific distribution of single survey data does not conform to the age distribution of birth cohorts to a large extent. The construction of hypothetical cohorts can help us convert the data on diseases of different aged cohorts that were born in the same year into a cohort that changes with age. Second, although some survey data can be traced, survey errors in a small range will lead to certain data errors. Using the imaginary queue method to correct the data can reduce the error.

Our basic method of constructing a hypothetical cohort was as follows: we obtained the age-specific mortality rate ${s}_{i}\left(t\right)$ in the survey year to construct a life table and a hypothetical cohort based on it. The survival of the elderly population ${p}_{i}\left(t\right), i= 65,\cdots ,105;t=2018$ at all ages is known. Taking the 65-year-old population in 2018 as the base number, the number of survivors in each age group older than 65 years is scaled according to the age-specific mortality rate in the life table.

The 65-year-old surviving population of the hypothetical cohort is set to ${P}_{65}={p}_{65}\left(2018\right)$, then traced ${p}_{66}\left(2018\right)$ back to 2017:

1 $${p}_{65}\left(2017\right)={p}_{66}\left(2018\right)/\left(1-{s}_{65}\left(2018\right)\right)$$

The scaling rate can be written as ${w}_{66}={p}_{65}\left(2018\right)/{p}_{65}\left(2017\right)$.

Similarly, we can extrapolate the population aged 67 to 105 in 2018 by tracing it back to the corresponding previous years. After obtaining the scaling rate, we scaled the cohort population in each disease state by rates to obtain data for each cohort that matched the scaled total population.

### Complex network model

The comorbidity of all chronic diseases increases exponentially as the number of chronic diseases increases. When the number of dimensions involved in all comorbidities is too high and the data dimension cannot meet the requirements of the analysis, it is necessary to select the most significant diseases among all comorbidities. The closeness of the comorbid relationship between diseases can be measured to some extent by frequency.

A complex network is a precise way to describe the complex relationships between entities in the physical world. Through network characteristics, we can accurately learn the relationships, community structure, hierarchy, and many other features of the actual network. In this study, we traverse each individual sample according to disease status and construct a complex network in which the nodes are involved.

Once we calculate the number of comorbid events between two kinds of chronic diseases, we give z values to measure the relative position in the overall sample data. The z score can be expressed as:

2 $${\text{z}}-{\text{score}}=\frac{x-\mu }{s}$$

In the complex network, x is represented by the weighted degree of each node. The possibility of emergency and frequency of comorbidity are considered comprehensively to determine which diseases have stronger and closer comorbidity connections. With a specific degree of dimensionality reduction as the goal, the z score can be used to select more connected multidimensional comorbid diseases.

### Multistate transition model

#### Assumptions

State transition probabilities can be calculated through consistent state transition tables based on birth cohort-specific and sex cohort-specific health and functional status variables obtained from cross-sectional data from survey rounds. Brunet and Struchiner constructed a continuous-time model for calculating nonparameters of incidence based on repeated cross-sectional survey data (Brunet & Struchiner, 1999) [23], and Goldman et al. (2004) [24] developed this model into a discrete time model. Megumi Kasajima introduced the model into the multistate transition model [25].

The above model is based on the following assumptions:

1. Disease processes involve absorption. All chronic diseases in this model are considered absorption states; i.e., there is no inverse process of recovery. This assumption is based on the pathological background that most chronic diseases do not have a complete cure;
2. The mortality rate in patients who had at least one disease was greater than the base age-sex mortality rate in patients with no comorbidities. Brunet and Struchiner considered the incidence of disease as a function of the difference between the prevalence at different times and the difference between disease-specific mortality and base mortality;
3. In association with the above assumptions, we believe that people who have a particular risk of disease mortality are considered part of the group of people that have already suffered;
4. Considering the limitations of the data and research methods, we assumed that the overall probability of death for an individual is the sum of the mortality of each individual suffering from disease and the base mortality. Of course, if the cause of death can be obtained under complex comorbidity conditions, this assumption will no longer be applicable.

#### Incidence calculation

To consider the joint distribution of disease states, a 2 × 2 joint table was constructed for each pair of diseases according to age, sex, and time period. This study evaluated new entry and exit points for each unit in a birth-sex 2 × 2 joint table to determine the incidence of each specific state.

For any disease i and disease j, a 2 × 2 joint table contains the initial population of each birth cohort (c) and the sex (s) at the beginning of the current moment t for four states $({d}_{i},{d}_{j})=(\mathrm{0,0}),(\mathrm{1,0}),(\mathrm{0,1}),(\mathrm{1,1})$, ${d}_{i}$ and ${d}_{j}$ indicate the state corresponding to the diagnosis of the disease. The population of each unit at the initial time t is expressed as ${pop}_{c,s,t}^{(i.j)}({d}_{i},{d}_{j})$.

In the interval between time t and t + 1, when the population of a cohort is reduced by a specific set unit, the cohort is regarded as a death cohort. For example, people in state $({d}_{i},{d}_{j})=(\mathrm{0,0})$ will only die from other factors and nonchronic diseases; therefore, the base mortality is reduced according to the base risk of death. Based on the same assumptions, the remaining three states had an additional risk of death from chronic disease.

The number of survivors at the end of t in each unit is expressed as ${surv}_{c,s,t}^{(i.j)}({d}_{i},{d}_{j})$:

3 $${surv}_{c,s,t}^{\left(i.j\right)}\left(\mathrm{0,0}\right)={pop}_{c,s,t}^{\left(i.j\right)}\left(\mathrm{0,0}\right)\times \left(1-{\alpha }_{base\left(c,s,t\right)}^{\left(i.j\right)}\right)$$

${\alpha }_{base(c,s,t)}^{(i.j)}$ indicates base mortality. The difference between the number of survivors and the initial number at t in units $({d}_{i},{d}_{j})=(\mathrm{0,1})$, $({d}_{i},{d}_{j})=(\mathrm{1,0})$ and $({d}_{i},{d}_{j})=(\mathrm{1,1})$ represents the base deaths plus the corresponding number of deaths from specific diseases. Therefore, the difference between the number of survivors at the end of t and the initial number of people at the beginning of t + 1 can be viewed as the number of disease transfer process cases, which requires accurate period-cohort data, which is why we constructed the hypothetical cohort.

For the group in state $({d}_{i},{d}_{j})=(\mathrm{0,0})$, there are two possible state transitions: suffering from disease i to state $({d}_{i},{d}_{j})=(\mathrm{1,0})$ and suffering from disease j to $({d}_{i},{d}_{j})=(\mathrm{0,1})$. Therefore, within the time interval t, the change in the number of units $({d}_{i},{d}_{j})=(\mathrm{0,0})$ can be regarded as the number of patients with disease onset i and the number of patients with disease onset j.

4 $${incidence}_{i\left(c,s,t\right)}^{\left(i,j\right)}\left(\mathrm{0,0}\right)+{incidence}_{j\left(c,s,t\right)}^{\left(i,j\right)}\left(\mathrm{0,0}\right)=\frac{{surv}_{c,s,t}^{\left(i,j\right)}\left(\mathrm{0,0}\right)-{pop}_{c,s,t+1}^{\left(i,j\right)}\left(\mathrm{0,0}\right)}{{pop}_{c,s,t}^{\left(i,j\right)}\left(\mathrm{0,0}\right)}$$

**Fig. 2:**
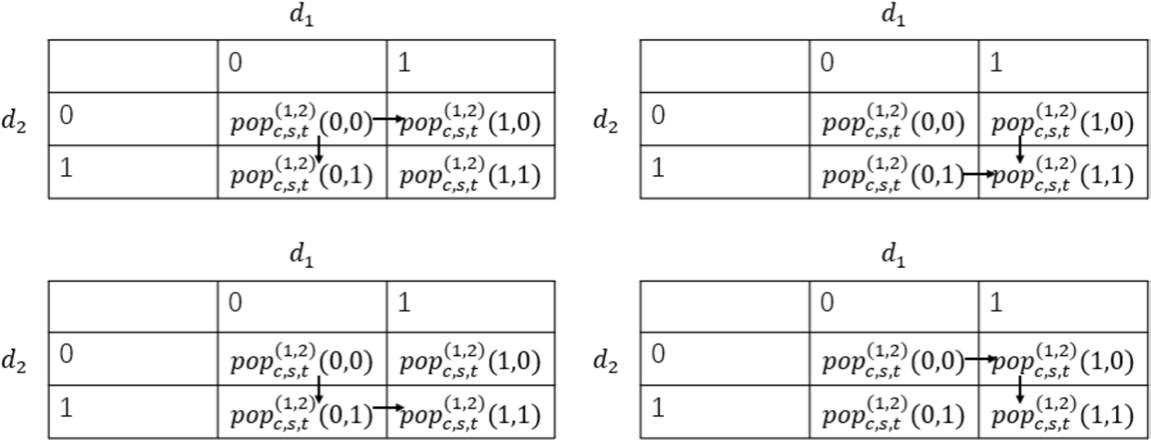
Multi-state transition process legend

In solving the transition probability of disease i and disease j from unit $\left({d}_{i},{d}_{j}\right)=\left(\mathrm{0,0}\right)$, in the 2 × 2 joint table shown in Fig. 2, we can clearly determine that the transfer process also affects the number of adjacent units. When $({d}_{i},{d}_{j})=(\mathrm{0,1}),$ is considered, the incidence of disease i will decrease ${pop}_{c,s,t}^{(i,j)}(\mathrm{0,1})$. Then, the number of transfers from units $({d}_{i},{d}_{j})=(\mathrm{0,1})$ and $({d}_{i},{d}_{j})=(\mathrm{1,0})$ can help us calculate the incidence of disease i and disease j as comorbidities, respectively.

When considering comorbidities, we converted the incidence calculated by the ${C}_{m}^{2}$ 2 × 2 joint table to a conditional incidence for m-dimensional status. By using the weighted average method, the incidence of disease *k* in multidimensional status $\left({d}_{1},{d}_{2},{d}_{3},\dots \dots ,{d}_{m}\right)$ can be calculated as follows:

5 $$\frac{\sum_{l\ne k \,{l=1,\dots ,m}}{incidence}_{k\left(c,s,t\right)}^{\left(k,l\right)}\left({d}_{k},{d}_{l}\right)\times {pop}_{c,s,t}^{\left(k,l\right)}\left({d}_{k},{d}_{l}\right)}{\sum_{l\ne k \,l=1,\dots ,m}{pop}_{c,s,t}^{\left(k,l\right)}\left({d}_{k},{d}_{l}\right)}$$

Table 2 shows the main variables used in this paper and their meanings.

**Table 2:** Main variables and corresponding explanations

Variable	Definition
${{\varvec{s}}}_{{\varvec{i}}}\left({\varvec{t}}\right)$	Death rate at time t by age i
${{\varvec{p}}}_{{\varvec{i}}}\left({\varvec{t}}\right)$	The number of people alive by age i at time t
${{\varvec{w}}}_{{\varvec{i}}}$	The scaling rate corresponding to age i in a hypothetical cohort
${{\varvec{p}}{\varvec{o}}{\varvec{p}}}_{{\varvec{c}},{\varvec{s}},{\varvec{t}}}^{({\varvec{i}}.{\varvec{j}})}({{\varvec{d}}}_{{\varvec{i}}},{{\varvec{d}}}_{{\varvec{j}}})$	Considering chronic diseases i and j at time t, the initial number of people in unit $({d}_{i},{d}_{j})$; c and s are used to represent age and sex groups
${\boldsymbol{\alpha }}_{{\varvec{b}}{\varvec{a}}{\varvec{s}}{\varvec{e}}({\varvec{c}},{\varvec{s}},{\varvec{t}})}^{({\varvec{i}}.{\varvec{j}})}$	The basic mortality of the (c,s) cohort at time t
${\boldsymbol{\alpha }}_{{\varvec{i}}({\varvec{c}},{\varvec{s}},{\varvec{t}})}$	Mortality of disease i of (c,s) cohort at time t
${{\varvec{s}}{\varvec{u}}{\varvec{r}}{\varvec{v}}}_{{\varvec{c}},{\varvec{s}},{\varvec{t}}}^{({\varvec{i}}.{\varvec{j}})}({{\varvec{d}}}_{{\varvec{i}}},{{\varvec{d}}}_{{\varvec{j}}})$	At the end t, the number of survivors of unit $({d}_{i},{d}_{j})$
${{\varvec{i}}{\varvec{n}}{\varvec{c}}{\varvec{i}}{\varvec{d}}{\varvec{e}}{\varvec{n}}{\varvec{c}}{\varvec{e}}}_{{\varvec{i}}\left({\varvec{c}},{\varvec{s}},{\varvec{t}}\right)}^{\left({\varvec{i}},{\varvec{j}}\right)}({{\varvec{d}}}_{{\varvec{i}}},{{\varvec{d}}}_{{\varvec{j}}})$	The incidence of disease i from $({d}_{i},{d}_{j})$

### Comorbidity analysis

When we consider m-dimensional chronic diseases associated with comorbidities, for each chronic disease, except for the conditions that have suffered from the target disease, ${d}_{i}=1$ all have $n={2}^{m}/2-1$ kinds of comorbidities, for which the incidence of the target disease is nonzero. Determining how to find several nonzero conditions far from the incidence in healthy patients requires further work that is based on the calculation of comorbidities, which is also how to analyze the incidence in patients with comorbidities.

The incidence of each chronic disease in patients with comorbidities changes differently with age and sex, and we cannot accurately express with which comorbidities the incidence of the target disease is highest. By determining the difference between the incidence of basic disease and the incidence of disease in healthy individuals, the superimposed effect of basic disease on the target disease can be determined. Therefore, to explore the difference in the incidence of the target disease in each disease state and the incidence of the target disease in the healthy state, we subtracted the incidence in the healthy state from the incidence rate in each comorbid state to obtain the difference sequence of incidence rates. The difference sequence values change with age, and there are *n* sequences corresponding to *n* comorbid states.

We observe whether the difference series has trends, cycles, etc., as the age of the cohort changes. When most of the difference series are stable time series at each age, we can generally use a constant, that is, the average of the differences, to estimate the total impact of basic diseases in comorbidities on the incidence of the target disease.

## Results

### Screening for major chronic diseases

**Fig. 3:**
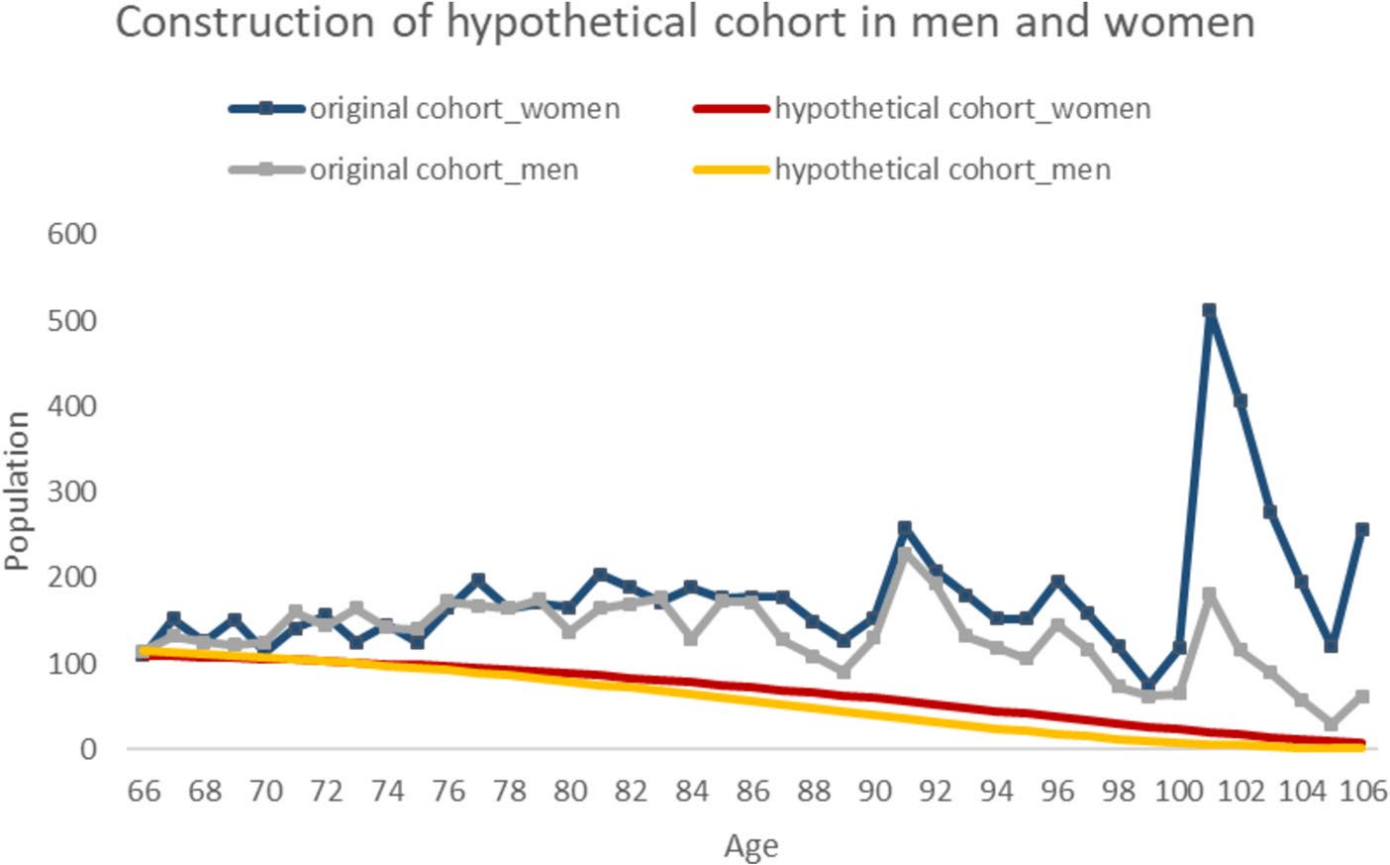
Hypothetical cohort and original cohort based on survey sample

Through normalization of the hypothetical cohort data, we obtained the change in the number of samples with age, as shown in Fig. 3.

**Fig. 4:**
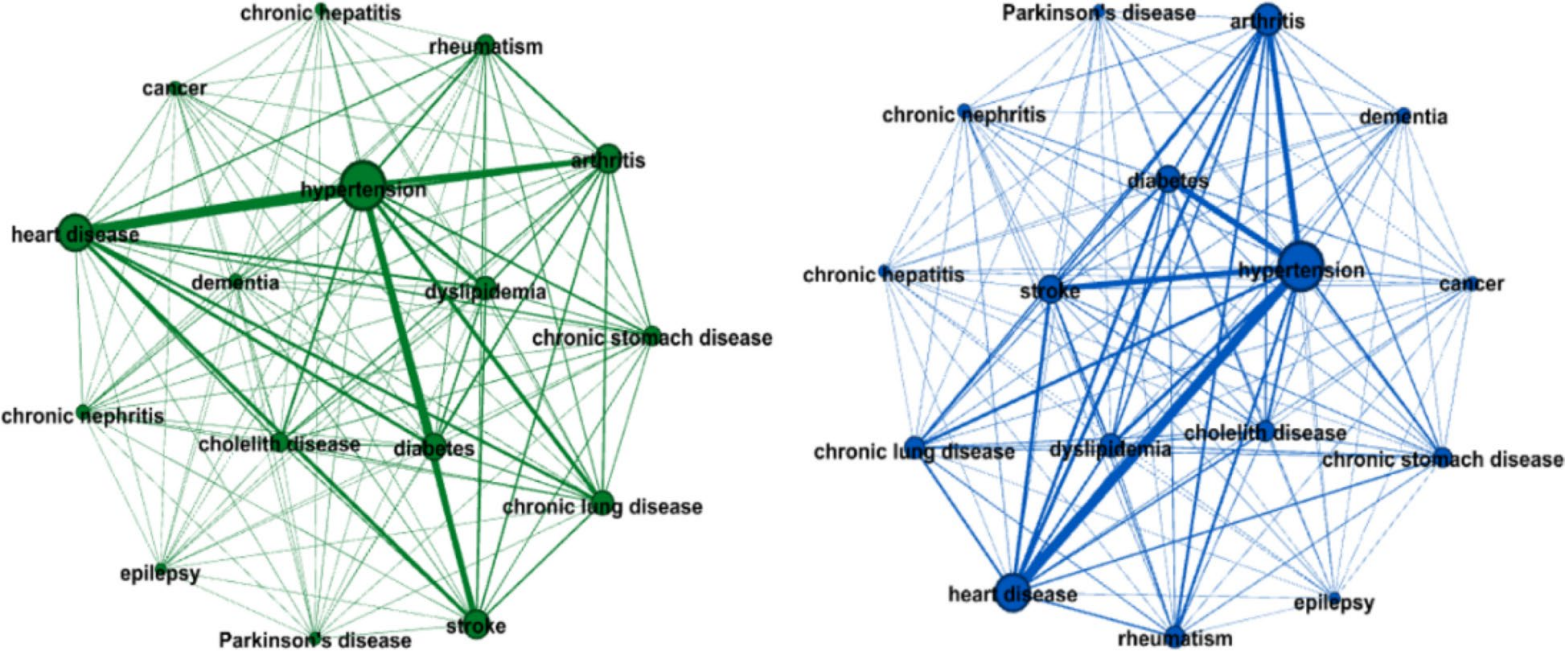
Complex network of chronic disease comorbidity in male and female cohorts

After traversing the disease state of each sample, if there are pairs of disease comorbidities, a comorbidity link edge is generated, and the disease comorbidity network is obtained as shown in Fig. 4 below. Through a complex network of dimensionality reduction, when the mean value of comorbidity of weighted degree was taken as the significance threshold (Table 3), we selected 7 chronic diseases, namely, hypertension, diabetes, heart disease, stroke, chronic lung disease, arthritis and dyslipidemia, that showed more significant comorbidity states when taking elderly men as the main research objects. Six kinds of chronic diseases were selected as the analysis objects for elderly women, namely, hypertension, diabetes, heart disease, stroke, arthritis and dyslipidemia. The comorbidity of chronic lung disease with other chronic diseases was more significant in elderly men than in elderly women.

**Table 3:** Weighted degree in a complex network of each disease

Label	Degree in male	z-score	Degree in female	z-score
Hypertension	5532	2.724	3217	2.673
Diabetes	2256	0.453	1310	0.427
Heart disease	3750	1.489	2246	1.530
Stroke	2543	0.652	1391	0.523
Chronic lung disease	1887	0.197	904	-0.051
Cancer	369	-0.855	218	-0.859
Chronic stomach disease	1132	-0.326	701	-0.290
Parkinson’s disease	215	-0.962	121	-0.973
Arthritis	2576	0.675	1705	0.893
Dementia	430	-0.813	275	-0.792
Epilepsy	115	-1.031	66	-1.038
Gallbladder disease	1191	-0.285	791	-0.184
Dyslipidemia	1726	0.086	1022	0.088
Rheumatism	1288	-0.218	868	-0.093
Chronic nephritis	467	-0.787	237	-0.836
Chronic hepatitis	163	-0.998	82	-1.038

### Incidence of major chronic diseases

Considering the 7 chronic diseases of elderly men, any pair of diseases can be constructed to calculate the incidence by sex and age, and the incidence of these 7-dimensional diseases under the condition of comorbidity can be weighted to obtain the following: There are two states, ${d}_{i}=0$ and ${d}_{i}=1$, for each chronic disease, corresponding to the 7-dimensional comorbidity $({d}_{1},{d}_{2},{d}_{3},{d}_{4},{d}_{5},{d}_{6},{d}_{7})$; in total, there are ${2}^{7}=128$ kinds of conditions, where the complete healthy state can be expressed as $\left({d}_{1},{d}_{2},{d}_{3},{d}_{4},{d}_{5},{d}_{6},{d}_{7}\right)=(\mathrm{0,0},\mathrm{0,0},\mathrm{0,0},0)$. The six chronic diseases considered by older women involved a total of 64 disease conditions.

#### Incidence in health status

**Fig. 5:**
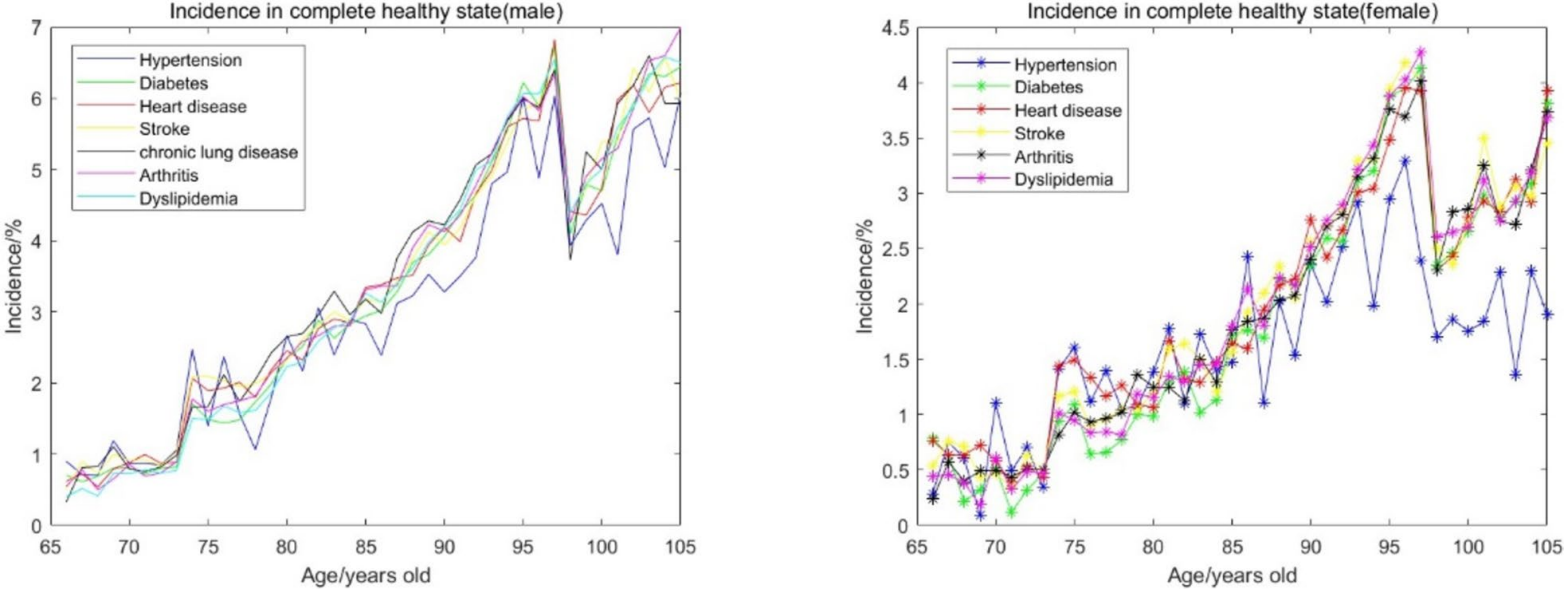
Incidence of various diseases in the state of complete health in men and women

We first selected the completely healthy state among the multiple comorbidity states for analysis. In Fig. 5, the smooth line shows the age-related incidence of 7 chronic diseases in elderly men, and the line with* shows the age-related incidence of 6 chronic diseases in elderly women.

**Fig. 6:**
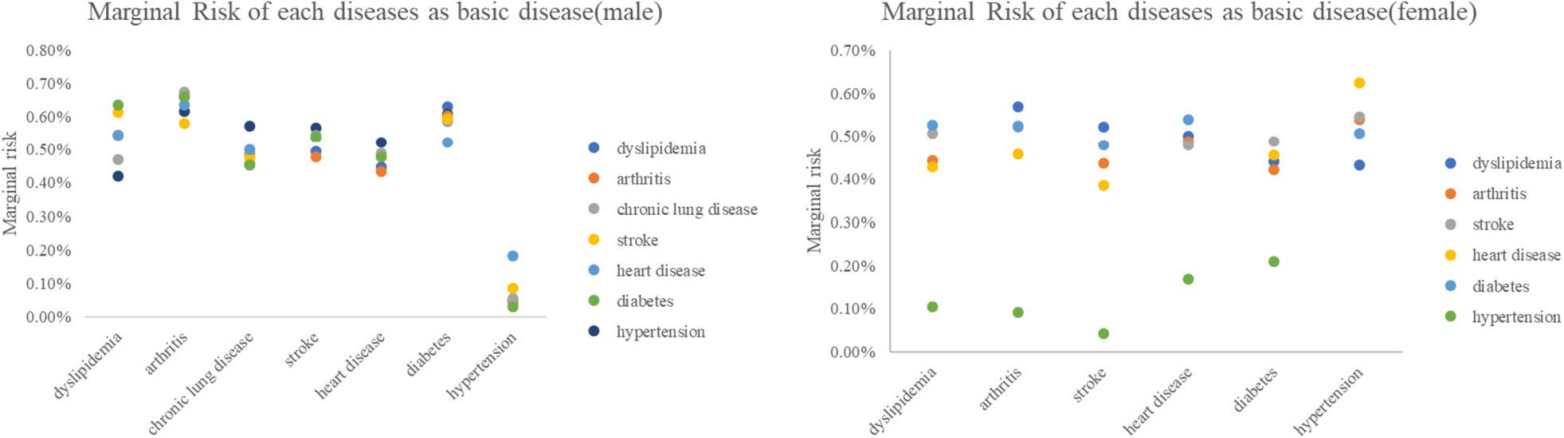
Marginal risk of each chronic disease as one basic disease on the incidence of other diseases

In a completely healthy state, the incidence of various chronic diseases increases with age, and the incidences of chronic diseases in men and women are relatively similar. However, it is worth noting that the incidence of almost all chronic diseases we screened out suddenly decreased around the age of 98 and then continued to increase, and this phenomenon was quite stable. Figure 6 also shows that on the basis of similar curve trends, the incidences of disease in men and women were different. The incidence of disease in male elderly people was greater than that in female elderly people in all age groups, and the incidence in male elderly people increased with age. In other words, the incidence of chronic diseases among elderly males is more significantly affected by age under healthy conditions. Moreover, the difference in the incidence of chronic diseases between men and women increases with age. The incidence is relatively close in the younger age range, and the gap increases in the older age range.

On the basis of the above incidence calculation, we can rank the risk of each disease in each age group: among them, the incidence of various diseases in the younger age group was increasingly varied. The risk of hypertension is significantly lower than that of other chronic diseases in individuals 90 years or older, and the risk of hypertension in elderly women decreases for those 95 years or older, which is significantly lower than that of other chronic diseases at the same age.

#### Incidence of comorbidities

When we analyzed the change pattern of the incidence of each target disease according to the form of comorbidities, we found that the incidence of the target disease significantly changed as the number of basic diseases increased. As the number of basic diseases increases, the increase in the incidence of target diseases affected by basic diseases does not show simple linear superposition but increases with the increase in the number of chronic diseases, showing a mutual influence amplification effect. The degree of amplification is based on the impact of each disease in combination with the basic disease combination as a single basic disease on the target disease.

When a basic disease is present, the added value of the incidence of the target disease ranges from 0.03% to 0.7%. When two basic diseases are present, the added value of the incidence of the target disease ranges from 0.4% to 1.6%. The positive effects of the three basic disease regimens on the incidence of the target disease were between 1.1% and 2.8%. The incidence of the four basic conditions increased between 2.3% and 5.2%. The percentage of patients with these five basic diseases was between 4.4% and 10.4%. The six basic diseases increased the incidence of the target diseases between 13 and 27%.

The seven chronic diseases analyzed in the elderly male population were hypertension, diabetes, heart disease, stroke, chronic lung disease, arthritis and dyslipidemia. Seven diseases were identified as the target diseases. Given that there are different numbers of basic diseases, the comorbidity condition with the largest difference between incidence in the diseased state and incidence in the healthy state was analyzed. The following two tables start with the single basic disease (Table 4 & 5). The contents in the previous column plus the contents in the corresponding table are expressed as the corresponding number of basic diseases that have the greatest impact. The value below is the magnitude of the marginal risk of the basic chronic disease to the target disease compared with the healthy state, that is, the increase in the incidence of the target disease when the basic chronic disease exists. In parentheses is 95% marginal risk 95% confidence interval. We can see that in the presence of a basic disease, patients with hypertension, diabetes, heart disease, chronic lung disease and dyslipidemia are most affected by arthritis; In the presence of basic diseases, the combination of arthritis and diabetes had the greatest impact on hypertension, chronic lung disease and dyslipidemia.

**Table 4:** The comorbidity combination of maximum promotion risk to chronic diseases in elderly men under different number of underlying diseases

	**One basic disease**	**Two basic diseases**	**Three basic diseases**	**Four basic diseases**	**Five basic diseases**	**Six basic diseases**
hypertension	arthritis 0.62% (0.60%,0.63%)	+ diabetes 1.48% (1.46%,1.49%)	+ chronic lung disease 2.77% (2.74%,2.80%)	+ stroke 4.82% (4.74%,4.89%)	+ heart disease 8.85% (7.69%,10.02%)	+ dyslipidemia 26.23% (24.34%,28.12%)
diabetes	arthritis 0.66% (0.64%,0.69%)	+ dyslipidemia 1.55% (1.54%,1.56%)	+ stroke 2.88% (2.85%,2.91%)	+ heart disease 5.11% (4.90%,5.33%)	+ chronic lung disease 10.40% (9.80%,11.00%)	+ hypertension 18.66% (17.95%,19.38%)
heart disease	arthritis 0.64% (0.63%,0.65%)	+ stroke 1.45% (1.42%,1.48%)	+ diabetes 2.60% (2.52%,2.71%)	+ chronic lung disease 4.83% (4.52%,5.14%)	+ dyslipidemia 10.39% (8.91%,11.87%)	+ hypertension 21.68% (21.23%,22.13%)
stroke	dyslipidemia0.61% (0.58%,0.65%)	+ diabetes 1.44% (1.35%,1.53%)	+ arthritis 2.74% (2.56%,2.91%)	+ chronic lung disease 4.96% (3.61%,6.31%)	+ heart disease 10.25% (8.96%,11.53%)	+ hypertension 18.33% (17.92%,18.74%)
chronic lung disease	arthritis 0.67% (0.66%,0.69%)	+ diabetes 1.53% (1.49%,1.56%)	+ stroke 2.85% (2.77%,2.92%)	+ heart disease 5.04% (3.29%,6.80%)	+ dyslipidemia 8.95% (7.39%,10.50%)	+ hypertension 13.80% (13.68%,13.93%)
arthritis	diabetes 0.60% (0.52%,0.68%)	+ chronic lung disease 1.34% (1.22%,1.46%)	+ stroke 2.46% (2.20%,2.71%)	+ dyslipidemia 4.36% (3.02%,5.69%)	+ heart disease 8.72% (8.05%,9.39%)	+ hypertension 15.61% (14.95%,16.27%)
dyslipidemia	arthritis 0.66% (0.64%,0.68%)	+ diabetes 1.57% (1.39%,1.76%)	+ stroke 2.84% (2.42%,3.26%)	+ chronic lung disease 5.08% (3.56%,6.60%)	+ heart disease 10.30% (8.92%,11.68%)	+ hypertension 19.96% (15.67%,24.25%)

The value in parentheses is the confidence interval of 95% of the corresponding marginal risk

**Table 5:** The comorbidity combination of maximum promotion risk to chronic diseases in elderly women under different number of underlying diseases

	**One basic disease**	**Two basic diseases**	**Three basic diseases**	**Four basic diseases**	**Five basic diseases**
hypertension	heart disease 0.62% (0.621%,0.629%)	+ stroke 1.50% (1.49%,1.51%)	+ arthritis 2.99% (2.96%,3.02%)	+ diabetes 6.30% (6.26%,6.33%)	+ dyslipidemia 26.07% (25.67%,26.47%)
diabetes	stroke 0.49% (0.486%,0.490%)	+ heart disease 1.21% (1.208%,1.215%)	+ dyslipidemia 2.47% (2.44%,2.51%)	+ arthritis 5.42% (3.96%,6.88%)	+ hypertension 8.35% (7.23%,9.48%)
heart disease	diabetes 0.54% (0.53%,0.56%)	+ dyslipidemia 1.35% (1.34%,1.36%)	+ stroke 2.97% (2.95%,2.99%)	+ arthritis 7.30% (7.21%,7.39%)	+ hypertension 12.83% (12.49%,13.17%)
stroke	dyslipidemia 0.52% (0.50%,0.54%)	+ diabetes 1.35% (1.32%,1.38%)	+ arthritis 2.85% (2.79%,2.91%)	+ heart disease 6.24% (6.06%,6.42%)	+ hypertension 10.36% (9.81%,10.92%)
arthritis	dyslipidemia 0.57% (0.569%,0.570%)	+ stroke 1.42% (1.39%,1.45%)	+ diabetes 3.06% (2.98%,3.15%)	+ heart disease 6.53% (5.56%,7.50%)	+ hypertension 9.69% (5.63%,13.75%)
dyslipidemia	diabetes 0.53% (0.52%,0.53%)	+ stroke 1.37% (1.36%,1.39%)	+ arthritis 2.88% (2.42%,3.35%)	+ heart disease 6.32% (4.38%,8.26%)	+ hypertension 9.84% (7.51%,12.17%)

The value in parentheses is the confidence interval of 95% of the corresponding marginal risk

The six major chronic diseases in elderly women were hypertension, diabetes, heart disease, stroke, arthritis and dyslipidemia. Similarly, we first analyzed the contributions of the remaining basic disease combinations to the incidence of each disease as a target disease. The target diseases that are most affected by different numbers of basic disease combinations are shown in the table below.

The above is the first analytical perspective that regards each chronic disease as a target disease; that is, the incidence probability of each target disease when not suffering from comorbid conditions is affected by the underlying disease. Under the influence of basic diseases, the incidence of various target diseases increases to varying degrees. Some chronic diseases generally have a greater growth rate, while others generally have a lower growth rate.

Based on the second analysis, if each target disease is already present, then the target disease exists as a basic disease, and we consider the impact of each target disease on the risk of other chronic diseases. Since the promoting effect of a combination of multiple basic diseases is based on the impact of one of the basic diseases, we can analyze its impact on comorbidities based on the impact of each disease.

We found that, in elderly men, the positive effect of hypertension, a basic disease, on other chronic diseases was significantly less pronounced than that of other chronic diseases. As a basic disease, arthritis has an average and slightly greater promoting effect on other chronic diseases.

Moreover, we found that when each chronic disease is considered the basic disease with the greatest impact on other chronic diseases, several combinations of chronic diseases with the greatest impact on each other appear (Fig. 6). These combinations show, to some extent, the degree of interaction between chronic diseases, reflecting the specific impact of each chronic disease on the other chronic diseases. Hypertension, a basic disease, has the greatest impact on heart disease, and heart disease, a basic disease, has the greatest impact on hypertension; dyslipidemia and diabetes are also related to each other.

In elderly women, arthritis, as a basic disease, no longer has a generally prominent impact on other diseases, but hypertension, as a basic disease, still has a significantly lower role in promoting target diseases than other chronic diseases. Hypertension and heart disease, as the basic diseases, are still the most common diseases. This further complements the contribution of each chronic disease to the other diseases. Taken together, these findings show that hypertension may not have a prominent impact on other chronic diseases under all comorbid conditions, but when it is a basic disease, heart disease will become a hidden disease that elderly people need to pay attention to.

## Discussion

As an important part of the elderly population, chronic diseases have attracted much attention, and the prevalence of chronic diseases can be roughly determined from previous surveys and censuses. How to further calculate the incidence of chronic diseases from the prevalence of chronic diseases is a problem we need to address. The multistate transition model provides a new perspective for us to calculate the incidence. We propose a combined model to better characterize the incidence of chronic diseases by sex and age.

With the model we constructed to calculate and analyze the disease incidence of chronic diseases with comorbidities, we can calculate the incidence of each disease under the combination of all comorbidities of chronic diseases when not suffering from the disease. When we select a chronic disease as the target disease, when it is not present, we analyze the impact of its incidence on the basic disease with the comorbidity; that is, the marginal risk of the basic disease. When it is present, the target disease exists as a basic disease during the analysis process, and we analyze the ranking of the remaining diseases affected by the target disease and observe which chronic disease the target disease has the greatest impact on.

In the process of building this model, we also have some aspects that we did not consider, which deserve further attention in the future. First, due to the limitations of the data sample and computing power, we cannot include all types of chronic diseases in our calculations and analyses, which leads us to ignore unmeasured diseases when calculating incidence rates and analyzing interactions. On the basis of the impact of a range of chronic diseases and the use of hypothetical cohorts, multiple rounds of survey samples can theoretically be used to explore the changes in incidence over time, and the methods can also complement each other by merging multiple datasets. This approach increases computational precision, but in this study, we used only single cross-sectional data points for age-dependent incidence rate calculations.

Furthermore, complex networks can essentially reflect the comorbid connections and interactions of chronic diseases. We hope to identify a more universal and systematic method for exploring diseases through similar higher-order complex networks or higher-order community structures. comorbidity patterns.

## Conclusions

According to our calculations and analysis of incidence, we found that during the process of comorbidity development, that is, in the presence of basic disease, the incidence of each chronic disease exhibited different characteristics from its incidence in a completely healthy state. Moreover, the incidences of different chronic diseases and comorbidities exhibit different developmental characteristics. These diseases are affected by basic conditions and have different effects on other chronic diseases. Among them, hypertension shows more prominent differences, which deserves further exploration of its incidence patterns. The incidence of disease in patients with comorbidities cannot be estimated uniformly with the incidence of disease in healthy individuals, and the correlation and interaction between them cannot be ignored.

## Data Availability

CHLHS datasets are available from the Peking University Open Research Data at https://opendata.pku.edu.cn/dataverse/CHADS;jsessionid=17f482b5f85f2719181be09c347b. The mortality of elderly in China obtained from the seventh census are available from National Bureau of Statistics at http://www.stats.gov.cn/sj/pcsj/rkpc/7rp/indexch.htm.
